# Influence of Molecular Noise on the Growth of Single Cells and Bacterial Populations

**DOI:** 10.1371/journal.pone.0029932

**Published:** 2012-01-06

**Authors:** Mischa Schmidt, Martin Creutziger, Peter Lenz

**Affiliations:** 1 Fachbereich Physik, Philipps-Universität Marburg, Marburg, Germany; 2 Zentrum für Synthetische Mikrobiologie, Philipps-Universität Marburg, Marburg, Germany; German Cancer Research Center, Germany

## Abstract

During the last decades experimental studies have revealed that single cells of a growing bacterial population are significantly exposed to molecular noise. Important sources for noise are low levels of metabolites and enzymes that cause significant statistical variations in the outcome of biochemical reactions. In this way molecular noise affects biological processes such as nutrient uptake, chemotactic tumbling behavior, or gene expression of genetically identical cells. These processes give rise to significant cell-to-cell variations of many directly observable quantities such as protein levels, cell sizes or individual doubling times. In this study we theoretically explore if there are evolutionary benefits of noise for a growing population of bacteria. We analyze different situations where noise is either suppressed or where it affects single cell behavior. We consider two specific examples that have been experimentally observed in wild-type *Escherichia coli* cells: (i) the precision of division site placement (at which molecular noise is highly suppressed) and (ii) the occurrence of noise-induced phenotypic variations in fluctuating environments. Surprisingly, our analysis reveals that in these specific situations both regulatory schemes [i.e. suppression of noise in example (i) and allowance of noise in example (ii)] do not lead to an increased growth rate of the population. Assuming that the observed regulatory schemes are indeed caused by the presence of noise our findings indicate that the evolutionary benefits of noise are more subtle than a simple growth advantage for a bacterial population in nutrient rich conditions.

## Introduction

In recent years it has become clear that many biological processes are intrinsically noisy leading to strong variations in composition and properties of individual cells belonging to the same population of genetically identical cells [1]. Important examples include the delay times in uptake of nutrients [2], variations in chemotactic tumbling behavior [3], entry into a dormant state [4], [5], [6], sporulation and competence [7], [8]. In many cases these variations are caused by noise at the transcriptional or translational level [1], [9], [10]. Studies in ecology and population genetics have shown that stochastic variability in phenotype can have an advantageous effect on populations growing in fluctuating environments. This effect is known as bet-hedging [11]. Typically, in these systems members of a population follow individual noise-induced strategies in preparation of environmental fluctuations. In particular, fluctuations in the transcription process might result in synthesis of proteins that are not required for growth in the given environment. Heterogeneous populations have been observed in different instances especially if the environmental fluctuations pose a severe danger for the population. An important example is that of entry into a dormant state [4], [5], [6]. During dormancy cells cannot grow, therefore, reducing the effective growth rate of the population. However, in hostile environments, for example if the population is exposed to antibiotics, the dormant cells do not die, and, thus, guarantee the survival of the population. In a similar way, a population can survive nutritional stress conditions by having some non-growing cells that sporulate [12].

Generally, the production of unneeded proteins leads to an additional burden reducing the growth rate [13], [14]. However, there are situations where this burden is compensated. For example, for a population growing in a fluctuating environment (with varying nutrients) synthesis of these additional proteins could be useful for the individual cells. It is *a priori* not clear if it is better to just produce the molecular machineries required to grow on the currently present nutrients or to produce additional machineries required for other (currently not present) nutrients. The first strategy has the advantage that the protein burden is lower thus leading to a higher growth rate for the current nutrient. However, the drawback is that after a shift in the medium (or if the current nutrient is running out) new molecular machinery required for growth has to be produced leading to a lag phase. This machinery possibly includes transporters, metabolic or catabolic enzymes and additional ribosomes. In the second strategy, there is no lag phase but the higher protein burden leads to a slower growth rate for all different nutrients. In fact, both strategies can be favorable depending on the timescale of the environmental fluctuations, the duration of the lag phase and the growth rates supported by the nutrients in the medium. As reviewed in [15], both strategies have been observed for *Escherichia coli*. For example, an *E. coli* population grown under glucose-limited conditions with a doubling time of 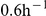 keeps growing without a lag-phase when transferred to a medium with excess of fructose, mannose, maltose, and ribose. However, a lag phase occurs when the population is transferred to a galactose or arabinose rich medium [16].

At the population level there can also be problems associated with phenotypic variations. For example, a specific phenotypic variation may increase the growth rate of individual cells but put the whole population in danger if too many cells have this phenotypic variation. In this case additional mechanisms are required to stabilize the population against this variation. An example is that of ‘cheater’ cells in yeast populations that do not express FLO1 [17]. This protein is essential for flocculation that guarantees the survival of populations in hostile environments that contain high concentrations of antimicrobials or ethanol by building up a protected space for the cells inside the floc. The cheater cells have a smaller protein burden and, thus, a higher growth rate. However, this phenotypic variation is not favorable for the population as a whole since cheater cells do not contribute to the flocculation process.

Phenotypic variations are also apparent in populations of *E. coli* growing in nutrient rich conditions. Although the macroscopic properties of bacterial populations are characterized by a few well-defined quantities such as growth rate, total cellular volume or mass, DNA content, and the number of ribosomes [18] the individual cells show large variations in birth size and even in individual interdivision times [19], [20]. Interestingly, there are also cellular processes that seem to be tightly regulated and that are not affected by phenotypic variations. An important example is cell division in *E. coli*. This process is precisely implemented and a mother cell divides into two daughter cells that differ at most by 3–10% in mass [19], [21], [22]. If this high precision is indeed a consequence of a tight regulation, an evolutionary advantage in cell division precision should be expected.

In this study we theoretically explore if there are evolutionary benefits of phenotypic noise for a growing population of genetically identical bacteria in non-hostile environments. In particular, we analyze if specific experimentally observed regulatory responses to noise (i.e. suppression or allowance of noise) are advantageous for a bacterial population growing in nutrient rich conditions. We consider two specific examples: (i) Division site placement in *E. coli* cells. In this process molecular noise is highly suppressed. We theoretically analyze if a precise implementation of the cell division process provides a growth advantage for a bacterial population. (ii) The occurrence of noise-induced phenotypic variations in fluctuating environments. Here, we address the question if it is favorable for a bacterial population to display phenotypic variations in a fluctuating environment, i.e. to form a heterogeneous population in which both of the above mentioned strategies are realized.

## Results

### Growth in non-fluctuating environments

We first analyzed the influence of single cell noise on exponentially growing bacterial populations in a homogeneous (non-changing) medium. As mentioned, there are many cellular processes that could be affected by the presence of noise. Here, we focused on its influence on the division process and we investigated if the growth rate of a bacterial population depends on the precision of cell division. In doing so we assume that an inexact cell division event produces two sister cells of unequal length and, consequently, of unequal mass. The smaller of the two daughter cells has less mass and thus less ribosomes, less transporter proteins and other molecular machinery needed to grow. We accordingly assume that it has a larger interdivision time (the time between birth and subsequent division) than its larger sister.

In our model the growth of a bacterial population is simulated by a sequence of cell division events. We start from one single newborn cell and simulate growth and division of this cell and its daughter cells. The individual doubling time 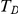 (the time a cell needs to double its mass) is set by the growth medium [18]. Because there are strong indications that cell division is coupled to mass [23] we assumed that the mass of the mother cell at cell division has a fixed value 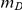 (that depends on the growth medium). In the absence of noise the mother cell, thus, divides into two daughter cells with birth mass 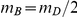. In this case, all cells have the same interdivision time given by the prescribed doubling time 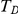. However, if the division process is noisy, the mother cell divides into two uneven daughter cells with mass 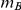 and 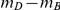. In the simulations 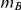 is given by a random number drawn from a normal distribution centered around 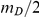 with standard deviation 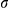 (for details see ‘Exp. methods’). Moreover, the two daughter cells of unequal size have different interdivision times. We consider only the case of limited lack of precision to make sure that both daughter cells contain a complete chromosome. To keep track of the division events in the population we used the “time until division” (tud) that represents the time left until an individual cell divides. At birth it is equal to the interdivision time and in every time step of the simulations, the tud of all cells is decreased by one (time unit). To calculate the tud distribution from the mass distribution we used the fact that the mass of an individual cell increases exponentially during its life cycle [24], [25]. Correspondingly, the interdivision time of a cell with birth math 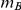 is given by 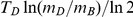. Thus, we implicitly assume that (under the nutrient-rich conditions considered here) the smaller daughter cell only needs longer to reach the doubling mass because of its smaller birth mass but does not have a growth defect, as has been experimentally observed in [26].

Before using this model to investigate how cell division noise affects the growth of the population, we first verified that in the absence of noise our model produces an exponentially growing population. To this end, we calculated an OD-plot by keeping track of the total mass of the population as function of time. Figure S1 clearly shows that the population indeed grows exponentially.

Similarly, we generated OD-plots for populations in the presence of divisional noise. The doubling time of the population 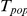 is obtained from the slope of the OD plot. Surprisingly, we found that 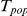 does not depend on the standard deviation of birth mass 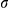 that quantifies the divisional noise, see Figure S2. Thus, noise in division site placement does not have any effect on the growth rate of the population.

**Figure 1:**
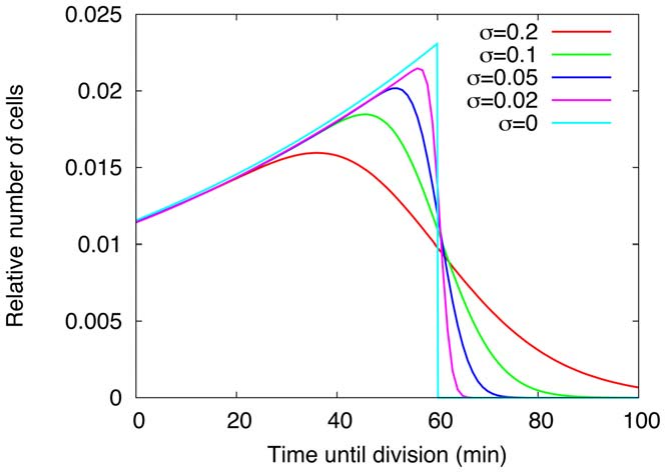
Steady state distributions of time until division for different noise levels. The steady state tud distributions 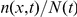 (where 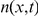 is the number of cells that have at time *t* a tud of *x* and 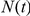 is the total number of cells at time *t*) were calculated from our model for different strength of divisional noise (quantified by the standard deviation 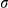 of birth masses of the daughter cells). Data shown are for 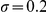 (red), 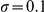 (green), 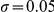 (blue), 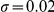 (magenta) and 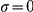 (cyan), where 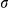 is given in units of division mass. The prescribed doubling time is 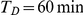 for all populations. For 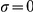 (cyan line) the distribution scales as 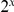.

To find the origin of this interesting behavior, we developed an analytical description of our numerical model. The key step is to identify a formula for the tud distribution of the growing population which can be obtained in a recursive fashion that relates the tud distributions at time 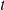 and 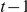


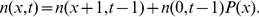
(1)Here, 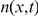 is the number of cells that have at time *t* a time until division of *x*. In particular, 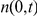 is the number of cells that divide at time *t*. 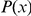 is the probability distribution for the interdivision times of the daughter cells that is obtained from the birth mass distribution, for details see Eq. (15) in ‘Exp. methods’. It is normalized to two, because every mother cell divides into two daughter cells. The last equation states that a cell that has a tud of *x* at time *t* either is a newborn cell or had a tud of 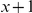 at time 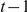. From Eq. (1) one can then show (as explained in detail in section 1 of Text S1) that the time evolution of the growing population obeys the following equation
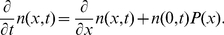
(2)Before analyzing Eq. (2) further we first tested if our method reproduces the results of other approaches [27]. To do so we calculated the steady state distribution 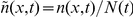 of the tud in the population. Here, 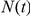 is the total number of cells. As shown in Fig. 1, in the absence of noise this distribution scales as 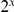 with the tud *x* (cyan line). In this case, the age *y* of a cell obeys 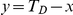, and our results are in agreement with the classical results on age distribution in growing bacterial populations [27]. As mentioned, 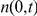 is the number of dividing cells and this quantity can be directly read off from the distribution of tud times. Fig. 1 shows that this quantity is independent of the standard deviation 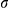.

Given this good agreement of our approach with classical results we proceeded with the theoretical analysis of Eq. (2) to analyze which ingredient of our model is responsible for the independence of 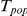 on 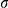. To do so we analyzed the time dependence of the total mass in the population
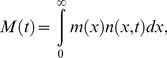
(3)which changes with time as
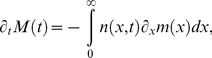
(4)as follows from Eq. (2) (for details see section 2 of Text S1). Upon using that individual cells increase their mass exponentially, i.e.
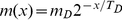
(5)one then finds
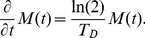
(6)The last equation shows that the change in total mass does not depend on the number of cells 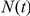 in the population. Thus, a population of cells with an exponential mass increase always grows exponentially with prescribed doubling time 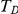 independently of how the total mass is partitioned between the different cells. For this reason the divisional noise does not affect the growth of the population. This analysis clearly demonstrates that the experimental observation that *E. coli* cells increase their mass exponentially is essential for our finding. For any non-exponential individual mass increase [entering via Eq. (5) into Eq. (6)] the total mass in the population depends on the number of cells leading to a dependence on the precision of cell division.

Given the surprising result that single cell division noise has no effect on the growth rate of a population we next asked whether noise affects other population observables. Many cellular quantities such as volume, mass, number of ribosomes proteins, or RNA content change during the cell cycle, presumably in an exponential manner [24], [25]. The distribution of all these quantities is easily calculated from the tud distribution. For example, for the volume one has
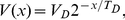
(7)where 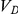 is the volume at cell division. Thus, the volume distribution is simply given by 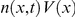 see Fig. S3. As one can see the tud (Fig. 1) and the volume distribution look quite similar, they only have a different scale on the x-axis. From this distribution one can easily calculate the mean volume and the standard deviation, see Fig. S4. As one can see these quantities have only a weak dependence on divisional noise. This is, of course, a consequence of the rather small differences in the tud distributions shown in Fig. 1 (for details see section 3 of Text S1).

### Growth in fluctuating environments

So far we have considered growth in homogeneous environments. In a next step we asked whether phenotypic variability provides growth advantages in fluctuating environments. To address this general question in a specific context we developed a theoretical model that describes the growth of a bacterial population in an environment with fluctuating supply of nutrients. More specifically, we consider a situation where the nutrients in the growth medium switch periodically (with period 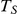). For simplicity we consider the case of a periodic switching between two limiting nutrients A and B.

There are mainly two strategies for how individual cells can cope with these changing conditions. One strategy (in the following referred to as strategy 1) is to synthesize only the molecular machinery required to grow on the nutrient that is currently present in the medium: if nutrient A is available only the machinery for growth on A is produced and the machinery required to grow on B is only produced if nutrient B is present. Thus, if the nutrient switches, new molecular machinery has to be synthesized. This requires an adaption time 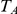 during which the cells do not grow. After adapting to the presence of B and absence of A cells grow with doubling time 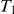. For simplicity we assume that the growth rate is identical for both growth conditions. However, our results do not depend on this specific assumption.

**Figure 2:**
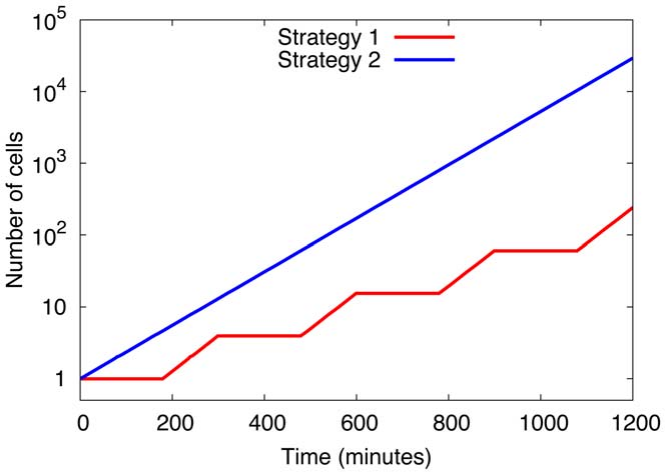
Growth curves of homogeneous populations in a fluctuating environment. Time dependence of the number of cells belonging to a homogeneous strategy 1 population (red curve) and a homogeneous strategy 2 population (blue curve). Data shown are for a switching time 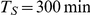, adaptation time 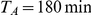, and doubling times 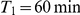 and 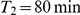. In this example, strategy 2 is advantageous due to the high value of 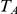.

A different strategy (in the following referred to as strategy 2) is to produce all molecular machinery to grow on A and B independent on whether A or B are currently present in the medium. In this way, no adaption is required after a switch in nutrients and the cells simply keep growing without a lag. Strategy 2 cells grow with doubling time 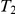 (again for both nutrients), and because of the extra-burden of producing non-needed proteins one expects 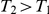 (i.e in the growth phase strategy 2 cells have a smaller growth rate than strategy 1 cells), see Fig. 2.

Both strategies have been observed experimentally in different situations, see Ref. [15] and references therein. We therefore first analyzed if our model reproduces the experimental findings that, depending on the values of the relevant parameters (adaption, doubling and switching time), different strategies are advantageous.

**Figure 3:**
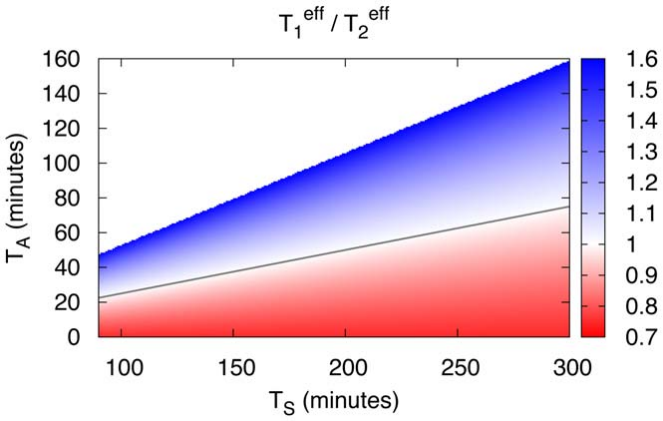
Optimal growth strategy for homogeneous populations. The ratio 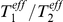 of doubling times of homogeneous populations growing with either strategy 1 or strategy 2 is shown for varying adaptation times 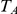 and switching times 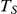. The effective doubling time 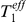 of the population growing with strategy 1 is given by Eq. (8), the effective doubling time 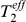 of the population growing with strategy 2 is given by Eq. (9). In the parameter range where 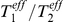 is larger than 1 (region shown in blue) a strategy 2 population is advantageous (i.e. the population with strategy 2 grows faster). In the region where 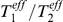 is smaller then 1 (shown in red), strategy 1 is advantageous. The grey line represents the phase boundary parameterized by Eq. (10). Data shown are for 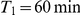 and 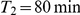. Here, and in the following Figs. only the range 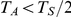, is shown. For higher values of 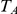, strategy 2 is trivially advantageous since strategy 1 cells stop growing as 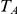 approaches 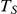.

To quantify this we determined the effective growth rate of homogeneous populations (exclusively consisting either of cells that use strategy 1 or strategy 2) in the fluctuating environment. For a strategy 1 population the effective doubling time (calculated by averaging over one period, see Fig. 2) is given by
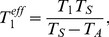
(8)while for a strategy 2 population one has
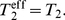
(9)
Fig. 3 shows the ratio 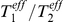 calculated from the last two equations as function of adaptation time 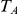 and switching time 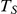. The blue region in Fig. 3 corresponds to the region in parameter space where this ratio is larger than 1 and in which the strategy 2 population grows faster, i.e. for these parameter values strategy 2 is advantageous over strategy 1. In contrast, strategy 1 is advantageous in the red regions of Fig. 3. The phase boundary between these two regions (shown as grey line in Fig. 3) is given by
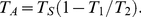
(10)As shown in Fig. 3, the strategy 2 population grows faster for long adaptation times and short switching times, i.e. when the ratio 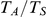 increases. More generally, both strategies can be advantageous depending on the adaptation time and the switching time. Thus, both growth strategies are only advantageous in a limited range of parameters 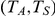. In a next step, we therefore asked if it might be favorable for a population to display phenotypic variations and form a population consisting of a noise-dependent mixture of strategy 1 and strategy 2 cells. The idea behind this is that with such a diversification a homogeneous population consisting of cells with, say, strategy 1 might be able to extend the parameter range for which it is advantageous by allowing some of its cells to convert to strategy 2. To test this possibility, we need to investigate if there are parameter values 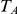 and 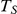 for which such a diversified population has the fastest growth.

**Figure 4:**
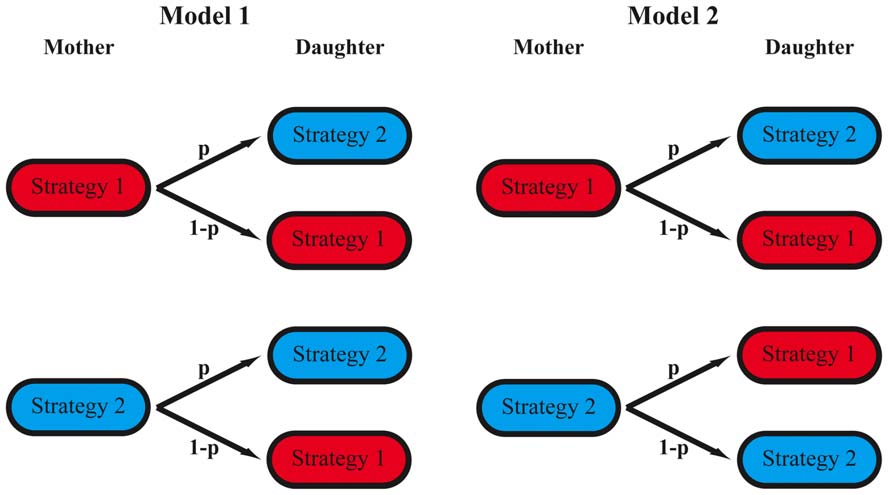
Division scheme in the two models for phenotypic diversification. A newborn cell randomly chooses a growth strategy. In model 1, strategy 2 is chosen with probability *p*, strategy 1 with probability 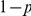 independent of the strategy of the mother cell. In model 2 this scheme is different, since here the probability of having strategy 1 or 2 depends on the strategy of the mother cell. Here, *p* denotes the probability that a newborn cell has a different strategy than its mother cell.

To analyze this, we implemented two different models for generating phenotypic variation as illustrated in Fig. 4. In both models, the individual cells are either growing with strategy 1 or with strategy 2. However, noise-induced fluctuations in gene expression can lead to a change of the growth strategy of the individual cells. For simplicity, we focus here on the case where such a change occurs only in newborn cells right after birth. This makes the following analysis easier but our conclusions do not depend on this assumption. We consider two scenarios. In the first scenario, described by model 1, only one of the two strategies is stable meaning that one strategy is predominantly chosen by a newborn cell. Here, the noise-induced fluctuations that lead to a change in growth strategy at birth are short-lived and are diluted out during one cell cycle. In the second scenario, described by model 2, both strategies are stable and the system can switch between the two strategies. In this case the noise-induced fluctuations are long-lived and the choice of growth strategy can be passed from the mother to the daughter cell. One possibility is to use an epigenetic inheritance mechanism [28]. As a consequence, the switching event in a cell has different effects on the growth strategy of its daughter cell in the two models: for example, consider a mother cell that has switched from strategy 1 to strategy 2. Then, in absence of any additional fluctuations the mother and its daughter cells have different strategies in model 1 but identical strategies in model 2.

Whenever a daughter cell has a different growth strategy than the mother cell, it has to adapt to the external conditions. This also requires an adaptation time, denoted by 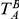. This adaptation process is either caused by the synthesis of additional metabolic machinery (for cells that switch from strategy 1 to strategy 2) or by the adjustment to a higher growth rate (for cells that switch from strategy 2 to strategy 1). For simplicity, we focus in the following on the special case where these two adaption times are equal: 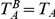. The influence of this specific assumption on our results is discussed below.

To formulate these models analytically it is again, as for the case of growth in homogeneous environments, convenient to use a continuum description. Then, one has at time *t*, 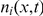 cells using growth strategy *i* with time until division *x*. At birth the interdivision time of a newborn cell with strategy *i* is drawn from a normal distribution 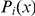 with mean 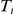 and standard deviation 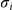 for 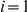 and 2. In the following, we use 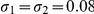 a typical value of cell division noise [19], [21], [22]. However, our general conclusions do not depend on this choice. By repeating the same analysis that led to Eq. (2) for two different distribution functions and by denoting the probability that a newborn cell has strategy 2 by *p* the time evolution equation for model 1 becomes
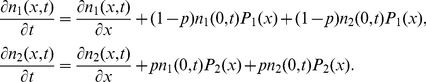
(11)In both equations, the first term on the right hand side describes the decrease of the tud of every cell with increasing time. The second term accounts for cell division events of cells with strategy 1 that create newborn cells with strategy 1 and 2 with probability 1−*p* and *p*, respectively. The third term accounts for the corresponding cell division events of cells with strategy 2. Because in this model there is no inheritance of growth strategy, the noise-induced fluctuations are diluted out after one generation and the probability *p* to have strategy 2 is independent of the strategy of the mother cell. In contrast, in model 2 the strategy choice of a newborn cell depends on the strategy of the mother. The probability that mother and daughter cells have the same strategy is 1−*p* (i.e. *p* is the probability that a newborn cell has a different strategy than its mother cell, see Fig. 4.). Correspondingly, for model 2 one has
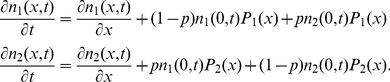
(12)In both models the total number of cells is given by
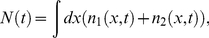
(13)from which the growth curve of a diversified population can be calculated. This allows the calculation of the doubling time 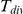 of the diversified population (by fitting this growth curve with an exponential).

**Figure 5:**
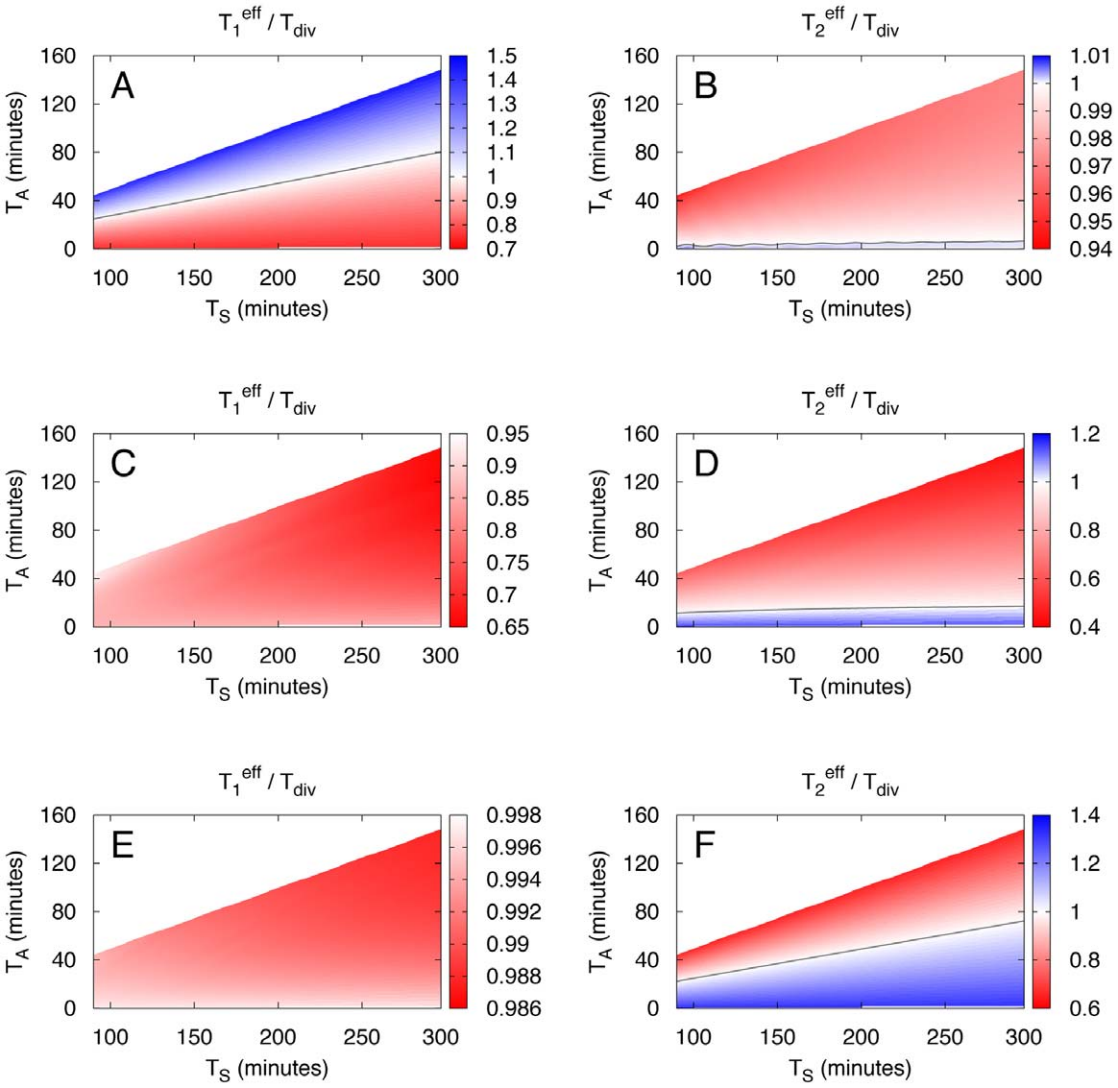
Comparison of doubling times of homogeneous populations and populations showing phenotypic variation according to model 1. The doubling time of a diversified population 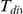 and a homogenous population are compared for different adaptation times 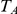 and switching times 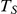. Single cell diversification is implemented according to model 1, see Fig. 4. Here the ratios 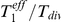 (left column) and 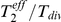 (right column) are shown. If this ratio is larger than 1 (shown in blue), the heterogeneous population grows faster. Data shown are for diversification probabilities 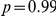 (A and B), 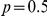 (C and D), and 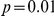 (E and F). In all cases the birth mass noise is 8% and data are only shown for 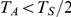 as in Fig. 3.

**Figure 6:**
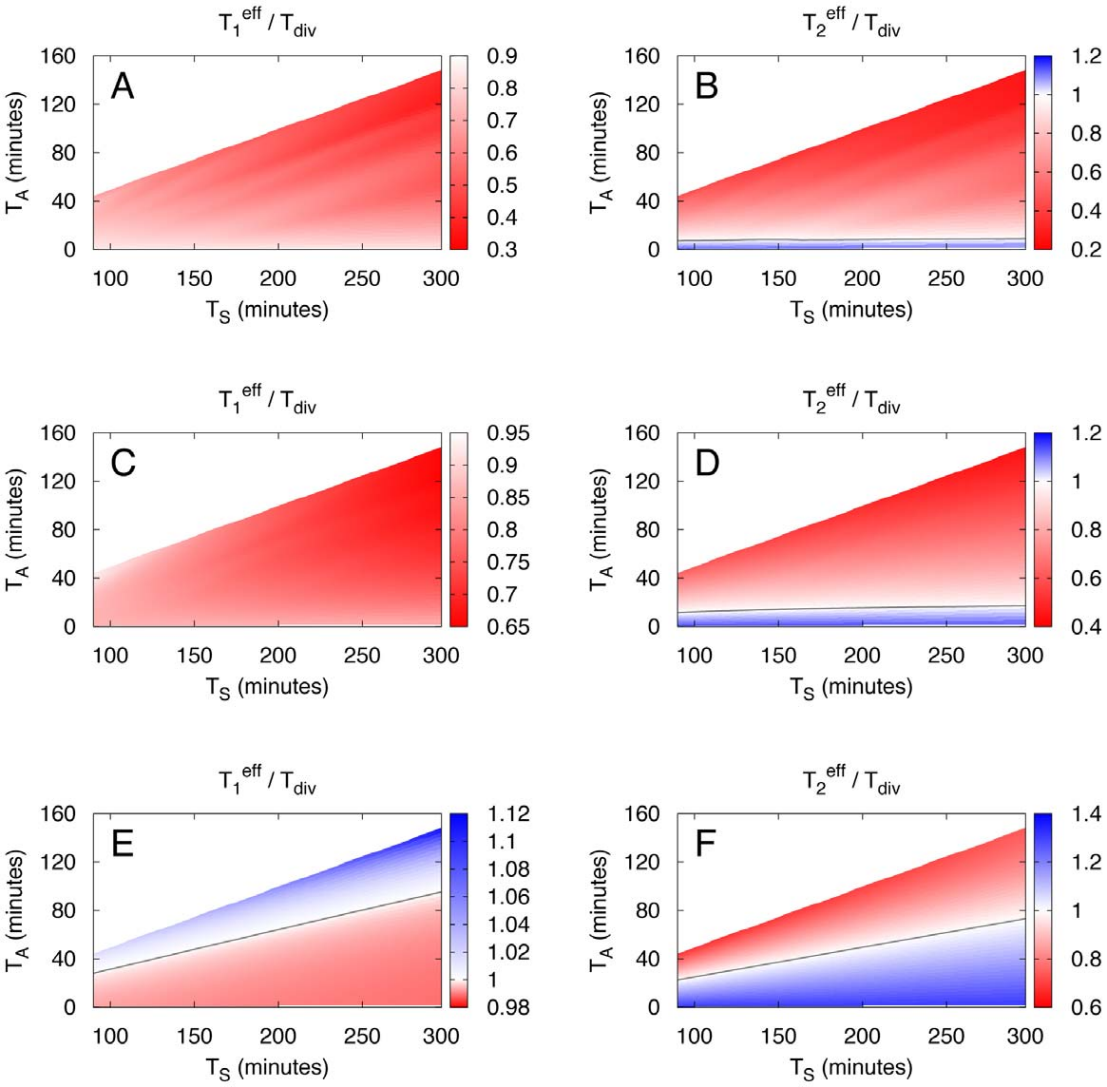
Comparison of doubling times of homogeneous populations and populations showing phenotypic variation according to model 2. The doubling times of a diversified population 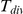 and a homogenous population are compared for different adaptation times 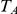 and switching times 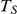. Single cell diversification is implemented according to model 2, see Fig. 4. Here the ratios 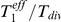 (left column) and 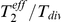 (right column) are shown. If this ratio is larger than 1 (shown in blue), the heterogeneous population grows faster. Data shown are for diversification probabilities 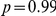 (A and B), 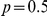 (C and D), and 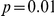 (E and F). In all cases the birth mass noise is 8% and data are only shown for 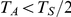 as in Fig. 3.

To determine whether phenotypic variation can provide a growth advantage in fluctuating environments we compared 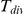 with the doubling times of the homogeneous populations by calculating 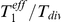 and 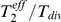. These quantities are shown as function of adaptation and switching time for different diversification probabilities *p* in Figs. 5 and 6. From inspection of these plots it becomes clear that a population displaying phenotypic variation can only grow faster than one of the homogeneous population (regions shown in blue), but never faster than both homogeneous populations. For example in model 1 with diversification probability *p* = 0.99, *T_A_* = 60 min and *T_S_* = 200 min the diversified population grows faster than a homogeneous strategy 1 population (see Fig. 5A). However, for the same parameter values a homogeneous strategy 2 population grows faster (see Fig. 5B). Such a behavior is found for all parameter values tested.

The conclusion that a homogeneous population is advantageous over a heterogeneous population under all conditions tested is surprising in light of earlier results [29],[30]. Several studies propose that phenotypic variation can be advantageous in fluctuating environments. To clarify which aspect of our model is responsible for this different conclusion, we looked for advantages of diversification under a variety of additional conditions.

As explained above, our model assumes periodic switching of the external conditions. We asked if our findings change in a more realistic scenario where 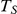 is not constant but varies randomly. To analyze this we implemented two different randomly switching environments, where the switching time is drawn from a normal distribution with mean 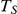 and from an exponential distribution with mean 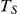, respectively. As shown in Figure S5 and S6, both of the “random switching environments” give rise to the same population doubling times 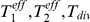 as the environment with periodic switching. This implies that the doubling times of all three populations, 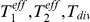 only depend on the average switching rate.

**Figure 7:**
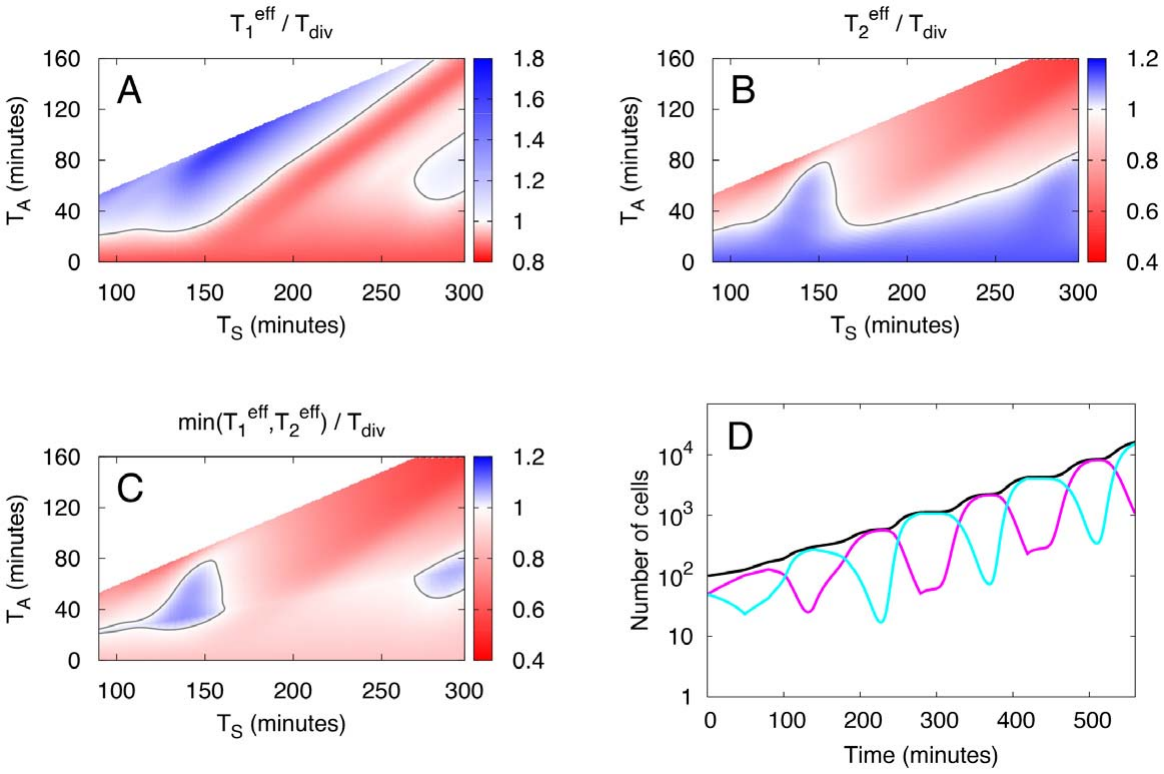
Population showing phenotypic variation according to model 2 growing without adaptation after a strategy switch. For 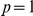 and 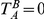, 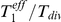 and 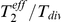 are shown as function of switching time 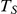 and adaptation time 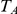 in Figs. A and B, respectively. Fig. C shows 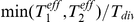 thus comparing the diversified population with the faster growing homogeneous population. In Fig. D the total number of cells 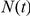 as function of time *t* is shown for the diversified population (black curve). The subpopulations growing with strategy 1 and 2 are shown in magenta and cyan, respectively. Data shown are for 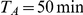 and 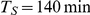. In all cases the birth mass noise is 8% and data are only shown for 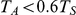.

An important feature of our model is that cells have to adapt when they have a different growth strategy than their mother cells. Above, we assumed that this adaptation process takes the same time as adaptation to an environmental switch, i.e. 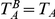. To analyze the influence of this assumption, we systematically varied the adaptation time 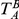. For 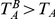, we find, as above, that a diversified population is always growing slower than one of the homogeneous populations, since in this case the growth rate of the diversified population is even further reduced. For decreasing 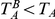, the doubling time of the diversified population 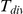 decreases continuously. As we are looking for situations where diversification is advantageous, we can focus on the case 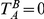. Systematic analysis showed that even in this case phenotypic diversification as described by model 1 does not provide a growth advantage. However, in model 2 diversification can be advantageous, but only for large diversification probabilities 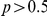, see Fig. 7. For example, for 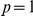, 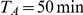 and *T_S_* = 140 min one has both 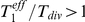 and 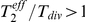 (i.e. this point in parameter space is blue in both Figs. 7A and 7B). However, as can be seen from Fig. 7C, the diversified population is only advantageous in a small region of phase space indicating that some fine-tuning of growth parameters 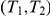 with environmental parameters 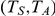 is required. For decreasing *p* the parameter range for which the diversified population is growing the fastest is getting smaller until it vanishes at 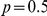.

## Discussion

In this study we address the question if the absence or presence of noise on the single cell level leads to evolutionary benefits for growing bacterial populations. It is well established that in hostile environments phenotypic noise provides an advantage for a bacterial population [31]. For example, as shown experimentally an *E. coli* population that is exposed to antibiotics survives by diversifying into a dormant subpopulation [6]. Here, however, we study evolutionary benefits of phenotypic noise for a growing population of genetically identical bacteria in non-hostile and nutrient-rich environments.

Under these conditions we find that generally noise at the single cell level has hardly any effect on the macroscopic properties of bacterial populations and neither affects its growth nor its composition (regarding e.g. mass and protein content). More specifically, we analyzed if suppression of divisional noise or allowance of noise in the transcriptional regulation of metabolic machineries leads to an increased growth rate of a population. Surprisingly, we found that in these scenarios the experimentally observed regulatory scheme (i.e. suppression of noise in division site placement and allowance of phenotypic variations for growth in fluctuating environments) does not lead to an increase in the growth rate of the population. While it is well established that molecular noise is essential for populations to cope with hostile situations that require random decision making (such as sporulation or entrance into competence or dormancy [4], [5], [6], [7], [8]) it is, as demonstrated here, difficult to identify an evolutionary relevant role of noise for bacterial growth in nutrient rich conditions. In fact, in the examples considered in this work, noise becomes only relevant for unrealistically high switching rates.

We found that divisional noise has no effect on the growth rate of a population of cells with exponential mass increase (such as *E. coli*). This finding is a direct consequence of the fact that (under the nutrient-rich conditions considered here) the growth rate of the individual cells is independent of cell size. Such a behavior has been observed experimentally in [26]. Of course, it cannot be excluded that under different conditions ‘non-linear’ effects become important. For example, one could imagine that in hostile environments smaller cells have a growth disadvantage making a symmetric cell division favorable. However, to be able to theoretically analyze such scenarios a systematic experimental characterization (along the lines of [26]) of the growth behavior of individual cells in hostile environments is required.

Given the fact that (in the scenarios considered) noise has no effect on the growth rate it is surprising that the Min system together with the nucleoid occlusion system, that determine the site of cell division in *E. coli*, shows such high precision with cell division occurring within 3%–10% (of total length) from mid-cell [19], [21], [22]. Z-ring formation is even more precise [32]. Because precise cell division does not result in faster growth the astonishing precision of cell division in *E. coli* is not the result of optimization of growth rate in nutrient-rich conditions. As mentioned, the precision could have some advantages under conditions different from those considered here. Another possibility is that the Min proteins simply keep the FtsZ ring from forming at the poles while the cell division site is determined by the action of nucleoid occlusion [33], [34], [35], [36]. In this way physical interactions associated with the position of the chromosomes would be responsible for division site placement. Such mechanisms where, e.g., the site of cell division is determined by the physical properties of the membrane and the associated turgor pressure have been intensively discussed in the literature [37], [38], [39].

Our results also indicate that for populations the presence of gene expression noise does not necessarily lead to evolutionary advantages at least not in the scenarios considered here, where such noise affects the metabolic program of individual cells in a fluctuating environment. We implemented two different models for generating phenotypic variation. Our model 1 describes short-term fluctuations that last only for one generation and are not inherited to the daughter cells. These fluctuations may originate from uneven partitioning of ribosomes or from improper sensing of the growth environment. Long-term fluctuations that persist for longer than one cell cycle are taken into account in our model 2 where the system can switch between the two growth strategies. Typical examples are bistable switches that are turned on or off by uneven partitioning of regulator proteins as explained in [1]. The phenotypic state of these switches can be inherited to the next generation [28].

Interestingly, for all (realistic) parameter values it is unfavorable to form a noise-induced mixture of cells with different strategies. Because at least one of the homogeneous populations is growing faster noise-induced diversification is unstable. These findings can be understood as follows: for given growth conditions either strategy 1 or strategy 2 is advantageous. Let's assume that, strategy 1 is advantageous. Then, the fastest growing population consists only of strategy 1 cells. For such a strategy 1 population diversification leads to formation of a subpopulation of cells with strategy 2. However, because strategy 2 cells grow (under the given conditions) slower than strategy 1 cells this diversification just implements the wrong strategy in some of cells leading to a decrease in growth rate of the population. For a strategy 2 population, however, diversification leads to an increase in growth rate since now some of the cells grow faster with strategy 1. In particular, this population grows the faster the larger the fraction of (diversified) strategy 1 cells is. Thus, in both cases the noise-induced fluctuations drive the system towards a homogeneous population with strategy 1. Similarly, for growth conditions that favor strategy 2 the noise-induced fluctuations drive the system towards a homogeneous strategy 2 population.

Thus, from an evolutionary point of view a diversified population is not stable: for populations with the better strategy every noise-induced fluctuation leading to a diversified population makes the population less fit and the fluctuations die out. Also for populations with the non-optimal strategy noise-induced fluctuations increase the growth rate. In this way the population is driven towards a homogeneous population. This finding does not depend on the details of the fluctuating environments and even holds for random switching (where now 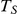 is a random time) between the two nutrients.

We found diversification to be favorable only for non-realistic conditions. Namely, in our model 2 for phenotypic diversification for a population that does not have to adapt to the change in growth strategy at birth 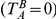 and that switches at high rates 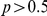. That this corresponds to a rather artificial growth strategy that also requires quite some fine-tuning of parameters can be made clear by considering the case 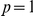. In this case all newborn cells have a different strategy than their mother cell. Let's consider the case where a switching event occurs at 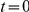. Then, all strategy 1 cells stop growing (due to adaptation) while strategy 2 cells keep growing and dividing. Because only strategy 2 cells divide and 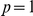 the fraction of strategy 1 cells increases while that of strategy 2 cells decreases. Thus, for appropriately chosen 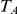 the population mainly consists of strategy 1 cells at 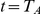. In this way large parts of the population grow with the higher doubling rate 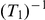 after the adaptation time is over. Thus, the diversification strategy optimizes both, the growth in the lag phase (by having a large fraction of strategy 2 cells for 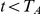) and the growth after adaption (by having a large fraction of strategy 1 cells for 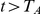). As growth proceeds the strategy 1 cells all divide giving rise to a population that mainly consists of strategy 2 cells. This leads to an oscillation of the composition of the population (see Fig. 7D) that alternates between the two homogeneous populations. Thus, the degree of diversification is not constant and in contrast to the other scenarios there are no stable subpopulations. It is also clear that this strategy only works if 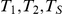 and 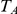 are chosen properly. In fact, the population can only increase its growth rate if it is able to anticipate the fluctuations in the growth medium and adjust its growth parameters accordingly. Such fine-tuning to environmental fluctuations can indeed lead to growth advantages as was shown experimentally for an engineered yeast strain in an accordingly chosen periodic environment [40]. However, since it involves parameter fine-tuning and unrealistic high switching rates we don't believe that this scenario has any relevance for real biological systems.

Our conclusion that diversification is (under realistic conditions) not advantageous in fluctuating environments is very different from those of other theoretical studies [29], [30]. However, we believe that their findings are the consequence of an incomplete comparison of all possible realizations of populations. In Ref. [29], the authors consider a population with two subpopulations growing at different growth rates. All cells can switch between the two subpopulations. In this model an environmental change simply leads to an exchange of growth rates of the subpopulations that immediately continue growing with the new doubling time. Thus, in contrast to our model the cells do not have to adapt to environmental changes. Diversification is declared favorable, if a population in which cells switch from the higher to the lower growth rate grows faster than a population in which only switching from low to high growth rate is allowed. However, this is only the case if the population anticipates the fluctuations and the cells switch into the state that will grow faster after the environment has changed.

In Ref. [30], the authors consider two types of heterogeneous populations. In one population, the cells choose the phenotype of highest growth rate under the current conditions by sensing the environment (responsive switching). In the other population, the cells choose their phenotype randomly (stochastic switching). A stochastic switching mechanism can lead to higher growth rate than a responsive switching mechanism if the switching rates between phenotypes follow the environmental changes. Thus, no equivalent of our strategy 2 population (of cells that do not switch phenotypes but grow with a burden) is considered.

In conclusion, these studies do not take into account that there are two reference populations (in our terminology a homogeneous strategy 1 and a homogeneous strategy 2 population) with which the diversified population has to be compared. Most of the results on advantageous effects of diversification are the consequence of this incomplete comparison with the reference populations. Furthermore, the models show that the strongest advantages occur for populations that are able to anticipate the fluctuations in the growth medium. As explained above, our model reproduces these growth advantages for anticipating populations if the adaptation process is neglected.

Our model is also applicable to more general situations than the simple switching between two nutrients. For example, our results and conclusions are not affected if a third nutrient C is present in between the other nutrients A and B provided that the strategy 2 cells do not produce the machinery required to grow on C and that both strategy 1 and strategy 2 cells need the same adaptation time. Thus, our model not only describes the switching sequences ABABAB but also ABCABC, ACBACB etc. and even scenarios with non-periodic occurrences of C.

Things become more interesting if for optimal growth on C a different strategy is required than for growth on A and B. But even then our conclusion that a homogeneous population is always favorable is not affected: both strategies lead to an effective doubling time over the full switching time. The effective doubling time of the mixture is then simply a linear superposition of the effective doubling times of the 2 homogeneous populations. For the case with two populations the optimal population then simply implements the strategy with the lowest effective doubling time. For more than 2 nutrients things are most interesting if the best solution is to regulate the metabolic machinery of sugar B in the presence of sugar A by one of the strategies and in the presence of sugar C by the other strategy. But even in this case it is not favorable to form a heterogeneous population because again the doubling time of the mixture is a linear combination of those of the homogeneous populations. Such a linear relation has its minimum at the boundaries, i.e. either at 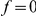 or 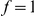 (where *f* is the fraction of the population with one of the two strategies). Thus, again the fastest growing population is a homogeneous one. The sum of doubling times during the intervals AB and CB determines whether it is strategy 1 or 2.

Given our main results that homogeneous populations always grow faster than a mixed one, it becomes clear that the observed variation in bacterial populations does not reflect an evolutionary advantage under the conditions considered here. We can only speculate here about its origin. For example, it could simply be of physical origin, namely that the costs for a more precise regulatory system exceed the benefit of being homogeneous. Or it might provide a mechanism to keep phenotypic variations alive that guarantee survival of the population under more severe or irreversible changes in the environment.

## Materials and Methods

The simulation of the growth of a bacterial population in a homogeneous environment started from a single newborn cell at time 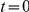 that divides at 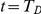. To determine when cells divide we keep track of the time until division *x* of every cell. A simulation step represents one time step and in every time step *x* is reduced by one for all present cells. At a given time all cells with 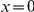 divide into two daughter cells. In the absence of noise, both daughter cells have the same birth mass 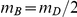 (where 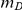 is the division mass of the mother cell) corresponding to an interdivision time of 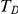. In the presence of noise, one daughter cell has birth mass 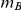 the other 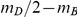, where 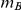 is drawn from a normal distribution with mean 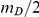 and standard deviation 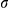, 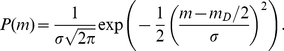
(14)Birth mass is transformed into the tud according to 
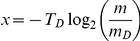
. Correspondingly, in Eqs. (2), (11) and (12) one has then
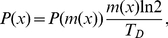
(15)where 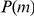 is given by Eq. (14) and 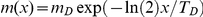.

The growth of populations in fluctuating environments is simulated by keeping track of the tud distributions 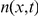. For a growing homogeneous population, the time evolution of the tud distribution is given by Eq. (2). Strategy 1 and strategy 2 populations behave like homogeneous populations except for the lag phase of strategy 1 populations during which the tud distribution is kept constant. The population doubling times are obtained by fitting the total number of cells in the population
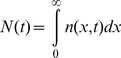
(16)at time *t* with an exponential.

The diversified populations grow according to Eqs. (11) and (12). Again, during the lag phase, the tud distribution of the strategy 1 subpopulation 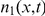 is kept constant. The population doubling time 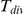 is obtained by fitting the total number of cells, Eq. (13), to an exponential.

For the simulations custom written C-programs were used.

## Supporting Information

Text S1 **Supporting text with derivation of equations.**
(PDF)

Figure S1
**OD-Plot of a growing population in the absence of divisional noise.** The OD plot was obtained by calculating the total mass of the population 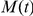 as function of time *t*. Mass is measured in units of the division mass 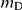, time *t* in units of generation time 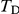. Population mass always doubles after one generation showing that the population indeed grows with prescribed doubling time 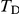.(TIF)

Figure S2
**Noise dependence of the population doubling time** 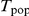 **.** The doubling time of the population 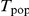 is obtained by fitting the total mass of the population 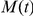 as function of time *t* to an exponential. 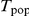 is shown in units of the prescribed doubling time 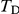 for different levels of birth mass noise quantified by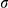.(TIF)

Figure S3
**Stationary volume distributions.** The volume histograms are shown for different strength of divisional noise: 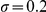
(red), 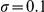
(green), 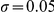
(blue), 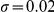
(magenta) and 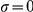
(cyan).(TIF)

Figure S4
**Influence of noise on the mean volume** 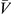 **and the standard deviation** 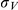 **of a population.** A: The mean volume (as given by Eq. 14 in Text S1) is measured in units of its value for 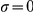. B: The standard deviation (calculated from Eq. 15 in Text S1) of the volume in units of its value for 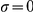.(TIF)

Figure S5
**Effect of noisy switching on phenotypic diversification described by model 1.** 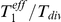 is shown as function of average switching time 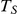 and adaptation time 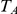. The diversification probability is 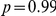. In figures A and B, the population growth was followed for 8 switching periods. In A, switching times are drawn from a normal distribution, in B from an exponential distribution. Figures C and D show averages over 100 runs shown in A and B, respectively. Birth mass noise is 8%. We only consider environmental switches with 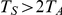.(TIF)

Figure S6
**Effect of noisy switching on phenotypic diversification described by model 2.** 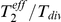 is shown as function of average switching time 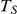 and adaptation time 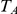. The diversification probability is 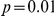. In figures A and B, the population growth was followed for 8 switching periods. In A, switching times are drawn from a normal distribution, in B from an exponential distribution. Figures C and D show averages over 100 runs shown in A and B, respectively. Birth mass noise is 8%. We only consider environmental switches with 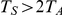.(TIF)

## References

[1] Smits WK, Kuipers OP, Veening JW (2006). Phenotypic variation in bacteria: the role of feedback regulation.. Nat Rev Microbiol.

[2] Megerle JA, Fritz G, Gerland U, Jung K, Radler JO (2008). Timing and dynamics of single cell gene expression in the arabinose utilization system.. Biophys J.

[3] Spudich JL, Koshland DE (1976). Non-genetic individuality: chance in the single cell.. Nature.

[4] Shah D, Zhang Z, Khodursky A, Kaldalu N, Kurg K (2006). Persisters: a distinct physiological state of *E. coli*.. BMC Microbiol.

[5] Levin BR (2004). Microbiology. Noninherited resistance to antibiotics.. Science.

[6] Balaban NQ, Merrin J, Chait R, Kowalik L, Leibler S (2004). Bacterial persistence as a phenotypic switch.. Science.

[7] Maughan H, Nicholson WL (2004). Stochastic processes influence stationary-phase decisions in *Bacillus subtilis*.. J Bacteriol.

[8] Veening JW, Smits WK, Hamoen LW, Kuipers OP (2006). Single cell analysis of gene expression patterns of competence development and initiation of sporulation in *Bacillus subtilis* grown on chemically defined media.. J Appl Microbiol.

[9] Elowitz MB, Levine AJ, Siggia ED, Swain PS (2002). Stochastic gene expression in a single cell.. Science.

[10] Ozbudak EM, Thattai M, Kurtser I, Grossman AD, van Oudenaarden A (2002). Regulation of noise in the expression of a single gene.. Nat Genet.

[11] Philippi T, Seger J (1989). Hedging Ones Evolutionary Bets, Revisited.. Trends in Ecology & Evolution.

[12] Errington J (1993). *Bacillus subtilis* sporulation: regulation of gene expression and control of morphogenesis.. Microbiol Rev.

[13] Koch AL (1983). The protein burden of lac operon products.. J Mol Evol.

[14] Dekel E, Alon U (2005). Optimality and evolutionary tuning of the expression level of a protein.. Nature.

[15] Kovarova-Kovar K, Egli T (1998). Growth kinetics of suspended microbial cells: from single-substrate-controlled growth to mixed-substrate kinetics.. Microbiol Mol Biol Rev.

[16] Lendenmann U, Egli T (1995). Is *Escherichia Coli* Growing in Glucose-Limited Chemostat Culture Able to Utilize Other Sugars without Lag.. Microbiology.

[17] Smukalla S, Caldara M, Pochet N, Beauvais A, Guadagnini S (2008). FLO1 is a variable green beard gene that drives biofilm-like cooperation in budding yeast.. Cell.

[18] Bremer H, Dennis PP, Neidhart FC (1996). Modulation of Chemical Composition and Other Parameters of the Cell by Growth Rate. *Escherichia coli* and *Salmonella*: Cellular and Molecular Biology 2nd ed.

[19] Koppes LH, Woldringh CL, Nanninga N (1978). Size variations and correlation of different cell cycle events in slow-growing *Escherichia coli*.. J Bacteriol.

[20] Harvey RJ, Marr AG, Painter PR (1967). Kinetics of growth of individual cells of *Escherichia coli* and *Azotobacter agilis*.. J Bacteriol.

[21] Trueba FJ (1982). On the precision and accuracy achieved by *Escherichia coli* cells at fission about their middle.. Arch Microbiol.

[22] Guberman JM, Fay A, Dworkin J, Wingreen NS, Gitai Z (2008). PSICIC: noise and asymmetry in bacterial division revealed by computational image analysis at sub-pixel resolution.. PLoS Comput Biol.

[23] Donachie WD, Begg KJ (1989). Cell length, nucleoid separation, and cell division of rod-shaped and spherical cells of *Escherichia coli*.. J Bacteriol.

[24] Ecker RE, Kokaisl G (1969). Synthesis of protein, ribonucleic acid, and ribosomes by individual bacterial cells in balanced growth.. J Bacteriol.

[25] Cooper S (1988). What is the bacterial growth law during the division cycle?. J Bacteriol.

[26] Schaechter M, Williamson JP, Hood JR, Koch AL (1962). Growth, cell and nuclear divisions in some bacteria.. Journal of general microbiology.

[27] Powell EO (1956). Growth rate and generation time of bacteria, with special reference to continuous culture.. J Gen Microbiol.

[28] Casadesus J, Low D (2006). Epigenetic gene regulation in the bacterial world.. Microbiology and Molecular Biology Reviews.

[29] Thattai M, van Oudenaarden A (2004). Stochastic gene expression in fluctuating environments.. Genetics.

[30] Kussell E, Leibler S (2005). Phenotypic diversity, population growth, and information in fluctuating environments.. Science.

[31] Davidson CJ, Surette MG (2008). Individuality in bacteria.. Annual review of genetics.

[32] Yu XC, Margolin W (1999). FtsZ ring clusters in min and partition mutants: role of both the Min system and the nucleoid in regulating FtsZ ring localization.. Molecular Microbiology.

[33] Woldringh CL, Mulder E, Huls PG, Vischer N (1991). Toporegulation of bacterial division according to the nucleoid occlusion model.. Res Microbiol.

[34] Yu XC, Margolin W (1999). FtsZ ring clusters in min and partition mutants: role of both the Min system and the nucleoid in regulating FtsZ ring localization.. Mol Microbiol.

[35] Margolin W (2001). Spatial regulation of cytokinesis in bacteria.. Curr Opin Microbiol.

[36] Errington J, Daniel RA, Scheffers DJ (2003). Cytokinesis in bacteria.. Microbiol Mol Biol Rev.

[37] Koch AL (2001).

[38] Koch AL, Holtje JV (1995). A physical basis for the precise location of the division site of rod-shaped bacteria: The Central Stress Model.. Microbiology-Sgm.

[39] Koch AL (2000). The bacterium's way for safe enlargement and division.. Applied and environmental microbiology.

[40] Acar M, Mettetal JT, van Oudenaarden A (2008). Stochastic switching as a survival strategy in fluctuating environments.. Nature genetics.

