# Supplementary material for: Influence of Molecular Noise on the Growth of Single Cells and Bacterial Populations
Source: PLoS One. 2012 Jan 6;7(1):e29932. doi: 10.1371/journal.pone.0029932 (PMC3253122; doi:10.1371/journal.pone.0029932)
Supplement: Text S1 — Supporting text with derivation of equations. (PDF) [file pone.0029932.s001.pdf]

Supporting information: Influence of molecular noise on the growth of single cells and bacterial populations

Mischa Schmidt and Martin Creutziger

Fachbereich Physik, Philipps-Universität Marburg, D-35032 Marburg, Germany

Peter Lenz

Fachbereich Physik, Philipps-Universität Marburg, D-35032 Marburg, Germany and
Zentrum für Synthetische Mikrobiologie,
Philipps-Universität Marburg, D-35032 Marburg, Germany

I. THE DYNAMICS OF THE TIME UNTIL DIVISION DISTRIBUTION

The properties of the population are described by the time until division (tud) distribution. At time t, $n(x,t)$ denotes the number of cells that will divide in x minutes. In particular, $n(0,t)$ is the number of cell division events at time t.

In every time step of the simulations, the tud x of every cell is reduced by one. Cells with $x = 0$ divide into two daughter cells. An inter division time (which is the tud at birth) drawn from the distribution $P(x)$ is assigned to the daughter cells. The tud distribution obeys the recursion relation

$$n(x,t+1) = n(x+1,t) + n(0,t)\,P(x)\,, \tag{1}$$

where $n(x,t)$ is the number of cells that have at time t a time until division of x. The last equation simply states that all cells that have a tud of x at time $t+1$ either had a tud of $x+1$ at time t or are newborn cells. Upon expanding $n(x,t+1) \simeq n(x,t) + \partial_t n(x,t)$ and $n(x+1,t) \simeq n(x,t) + \partial_x n(x,t)$ Eq. (1) becomes

$$\partial_t n(x,t) = \partial_x n(x,t) + n(0,t)\,P(x)\,, \tag{2}$$

where $\partial_x \equiv \partial/\partial x$ and $\partial_t \equiv \partial/\partial t$. This differential equation describes the dynamics of the tud distribution.

II. MASS GROWTH OF A POPULATION

As explained in the previous section, the dynamics of the tud distribution is given by Eq. (2). At time t, the population consists of $N(t)$ cells with total mass $M(t)$ with

$$N(t) = \int_0^\infty n(x,t)\,dx, \tag{3}$$

and

$$M(t) = \int_0^\infty m(x)\,n(x,t)\,dx, \tag{4}$$

where $m(x)$ is the mass of a cell with tud x. Upon differentiating the last equation and using Eq. (2) one obtains

$$\partial_t M(t) = \int_0^\infty m(x)\left(\partial_x n(x,t) + n(0,t)\,P(x)\right)dx \tag{5}$$

$$= \left[m(x)\,n(x,t)\right]_0^\infty - \int_0^\infty n(x,t)\,\partial_x m(x)\,dx + \int_0^\infty n(0,t)\,m(x)\,P(x)\,dx \tag{6}$$

$$= -m_D n(0,t) - \int_0^\infty n(x,t)\,\partial_x m(x)\,dx + n(0,t)\int_0^\infty m(x)\,P(x)\,dx. \tag{7}$$

Here, $m_D = m(0)$ is the division mass of the cells. The last term of Eq. (7) can be simplified by using

$$P(x) = 2\int_0^{m_D} \delta\left(x - x(m)\right)P(m)\,dm, \tag{8}$$

where $\delta(x)$ is the Dirac delta function. Note, $P(x)$ is normalized to two, since every cell division event produces two newborn cells. With the last identity the

last term of Eq. (7) becomes

$$\int_0^\infty m(x)\, P(x)\, dx = 2\int_0^{m_D} mP(m)\, dm = m_D. \tag{9}$$

Therefore, Eq. (7) simplifies,

$$\partial_t M(t) = -\int_0^\infty n(x,t)\, \partial_x m(x)\, dx. \tag{10}$$

The last equation is Eq. (4) in the main text. By taking into account that the single cells increase their mass exponentially, i.e.

$$m(x) = m_D 2^{-x/T_D}, \tag{11}$$

Eq. (10) becomes

$$\partial_t M(t) = \frac{\ln(2)}{T_D} M(t). \tag{12}$$

III. POPULATION AVERAGES

Here we discuss the influence of single cell noise on population observables. Every cell in the population has a property $V(x)$ (for example the volume), that depends on its time until division x. During the growth cycle, $V(x)$ grows exponentially, i.e.

$$V(x) = V_D 2^{-x/T_D}, \tag{13}$$

with $V_D = V(0)$, the volume at cell division. Figure S3 shows the volume distribution for different divisional noise levels quantified by σ.

The population average and the variance of $V(x)$ are given by

$$\overline{V} = \frac{1}{T_D}\int_0^{T_D} dt \int_0^\infty \tilde{n}(x,t)\, V(x)\, dx \tag{14}$$

and

$$\mathrm{Var}(V) = \int_0^\infty \tilde{n}(x,t) \left(V(x) - \overline{V}\right)^2 dx, \tag{15}$$

respectively. Here,

$$\tilde{n}(x,t) = \frac{n(x,t)}{N(t)}, \tag{16}$$

where $N(t)$ is the total number of cells in the population (see eq. (3)).

First, we consider the case of cell division without noise, where

$$\tilde{n}(x,t) = \delta(x-t). \tag{17}$$

Substitution into Eq. (14) yields

$$\overline{V} = \frac{1}{T_D} \int_0^{T_D} V_D 2^{-t/T_D} dt = \frac{1}{2\ln(2)} V_D. \tag{18}$$

Now we want to compare this with the population average in the presence of noise. As seen in Fig. 1, when considering very small (but non-zero) variations in interdivision times, the relative tud distribution can be approximated by

$$\tilde{n}(x,t) = \frac{\ln(2)}{T_D} 2^{x/T_D} \left(1 - \theta(x - T_D)\right), \tag{19}$$

where, $\theta(x)$ is the Heaviside step function. In this case, the population average is:

$$\overline{V} = \frac{\ln(2)}{T_D} V_D \int_0^{T_D} dx = \ln(2) V_D. \tag{20}$$

The variance of $V(x)$ is given by

$$\mathrm{Var}(V) = (V_D)^2 \left(0.5 - (\ln(2))^2\right). \tag{21}$$

To test the impact of noise on the population observable, we performed simulations to determine $\overline{V}$ from Eq. (14) for different noise levels σ. The results are shown in Figure S4A. It can be seen that the noise dependence of $\overline{V}$ is weak. In addition, we used Eq. (15) to calculate the standard deviation of the volume

$$\sigma_V = \sqrt{\mathrm{Var}(V)}. \tag{22}$$

The results are shown in figure S4B. It can be seen that σ_V of the population is approximately equal to the standard deviation of the divisional noise σ.
