# Supplementary figures and images for: Influence of Molecular Noise on the Growth of Single Cells and Bacterial Populations
Source: PLoS One. 2012 Jan 6;7(1):e29932. doi: 10.1371/journal.pone.0029932 (PMC3253122; doi:10.1371/journal.pone.0029932)

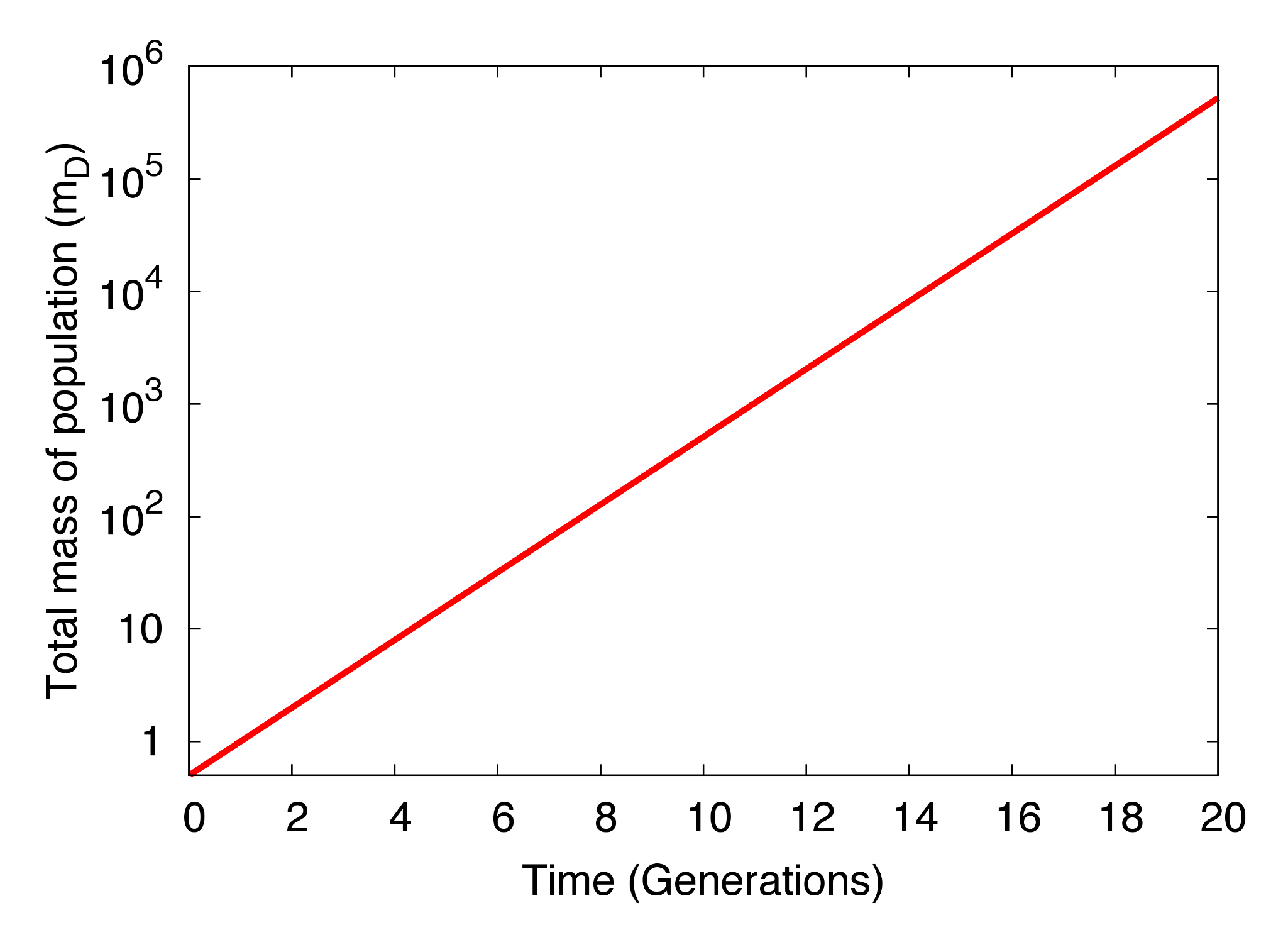

Supplement: Figure S1 — OD-Plot of a growing population in the absence of divisional noise. The OD plot was obtained by calculating the total mass of the population as function of time t. Mass is measured in units of the division mass , time t in units of generation time . Population mass always doubles after one generation showing that the population indeed grows with prescribed doubling time . (TIF) [file pone.0029932.s002.tif]

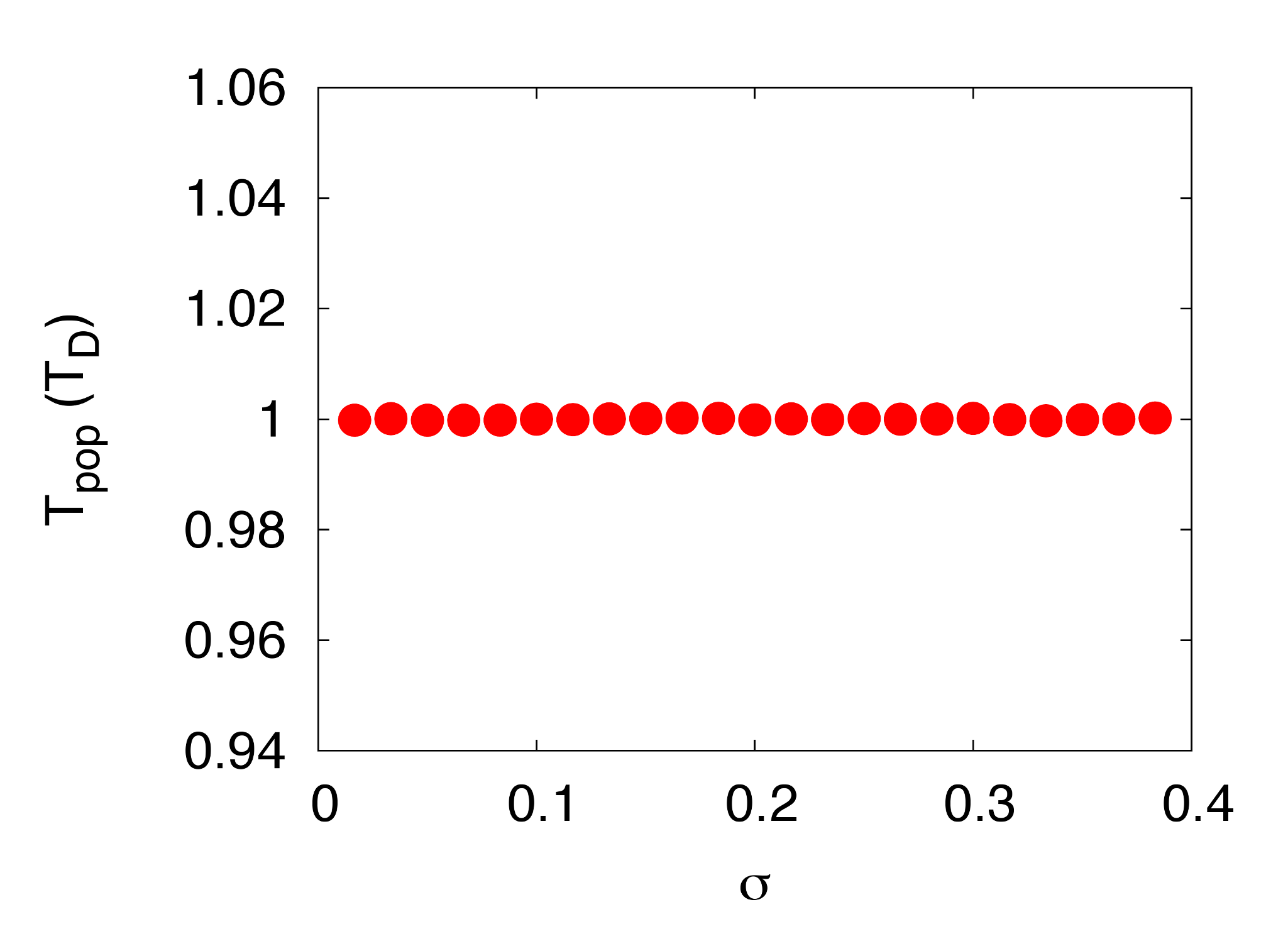

Supplement: Figure S2 — Noise dependence of the population doubling time . The doubling time of the population is obtained by fitting the total mass of the population as function of time t to an exponential. is shown in units of the prescribed doubling time for different levels of birth mass noise quantified by. (TIF) [file pone.0029932.s003.tif]

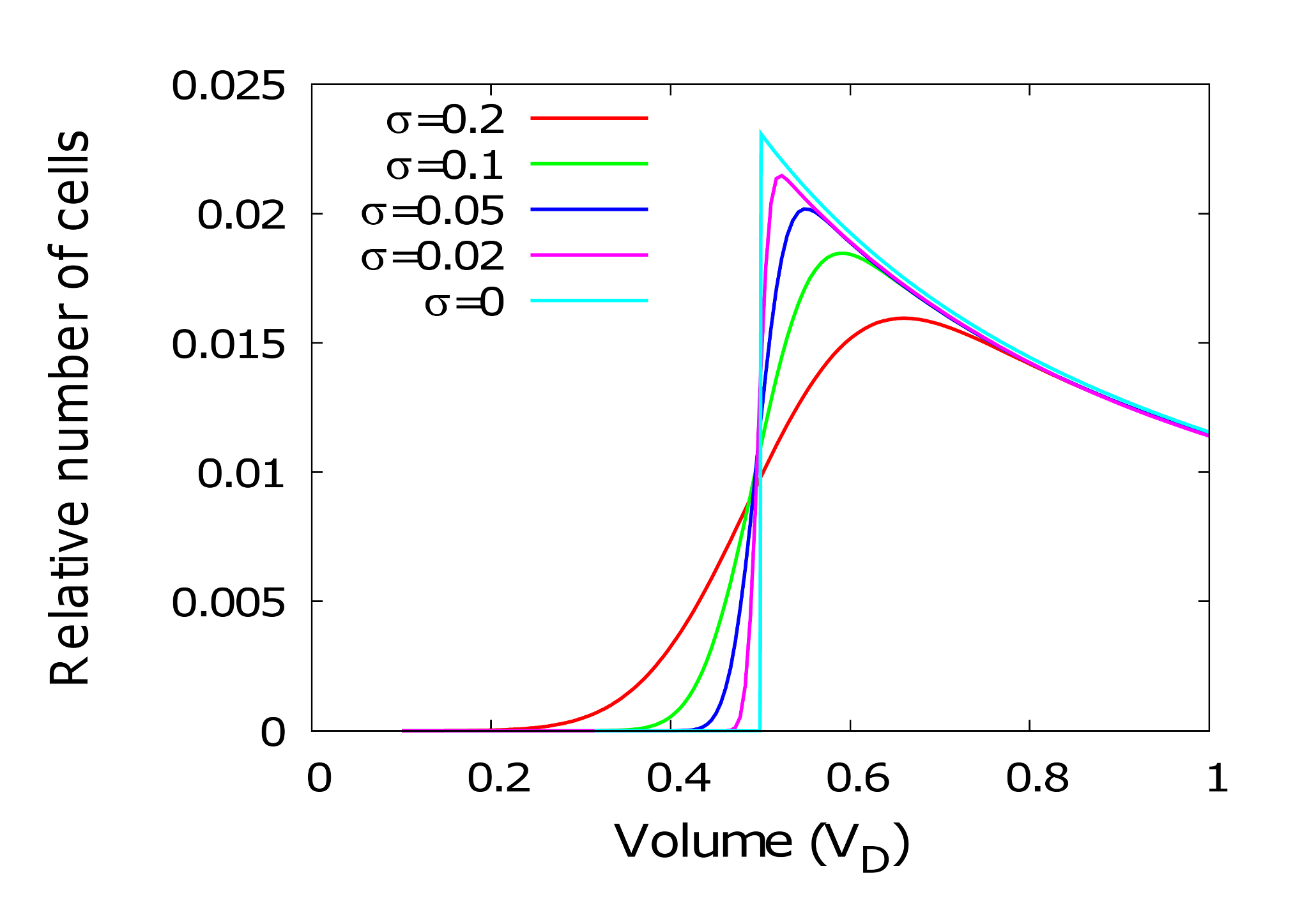

Supplement: Figure S3 — Stationary volume distributions. The volume histograms are shown for different strength of divisional noise: (red), (green), (blue), (magenta) and (cyan). (TIF) [file pone.0029932.s004.tif]

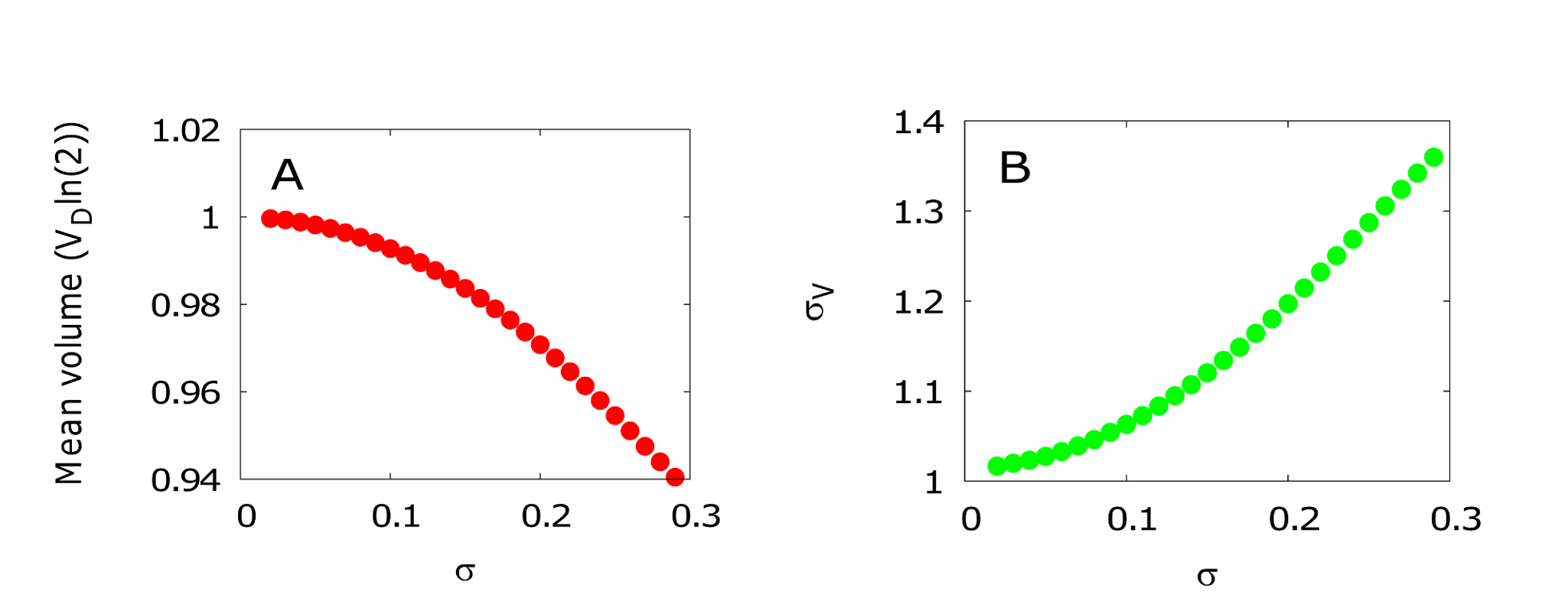

Supplement: Figure S4 — Influence of noise on the mean volume and the standard deviation of a population. A: The mean volume (as given by Eq. 14 in Text S1) is measured in units of its value for . B: The standard deviation (calculated from Eq. 15 in Text S1) of the volume in units of its value for . (TIF) [file pone.0029932.s005.tif]

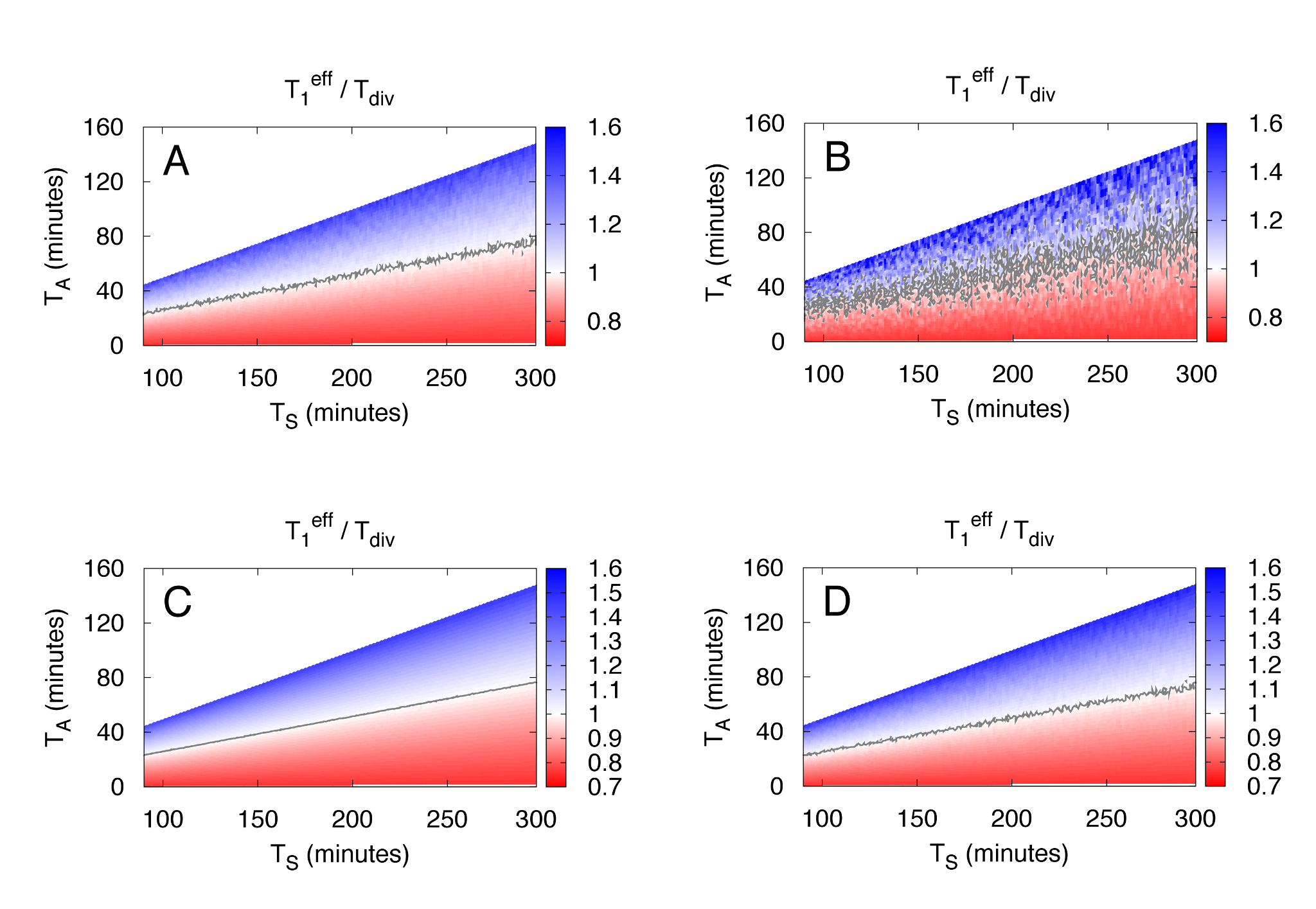

Supplement: Figure S5 — Effect of noisy switching on phenotypic diversification described by model 1. is shown as function of average switching time and adaptation time . The diversification probability is . In figures A and B, the population growth was followed for 8 switching periods. In A, switching times are drawn from a normal distribution, in B from an exponential distribution. Figures C and D show averages over 100 runs shown in A and B, respectively. Birth mass noise is 8%. We only consider environmental switches with . (TIF) [file pone.0029932.s006.tif]

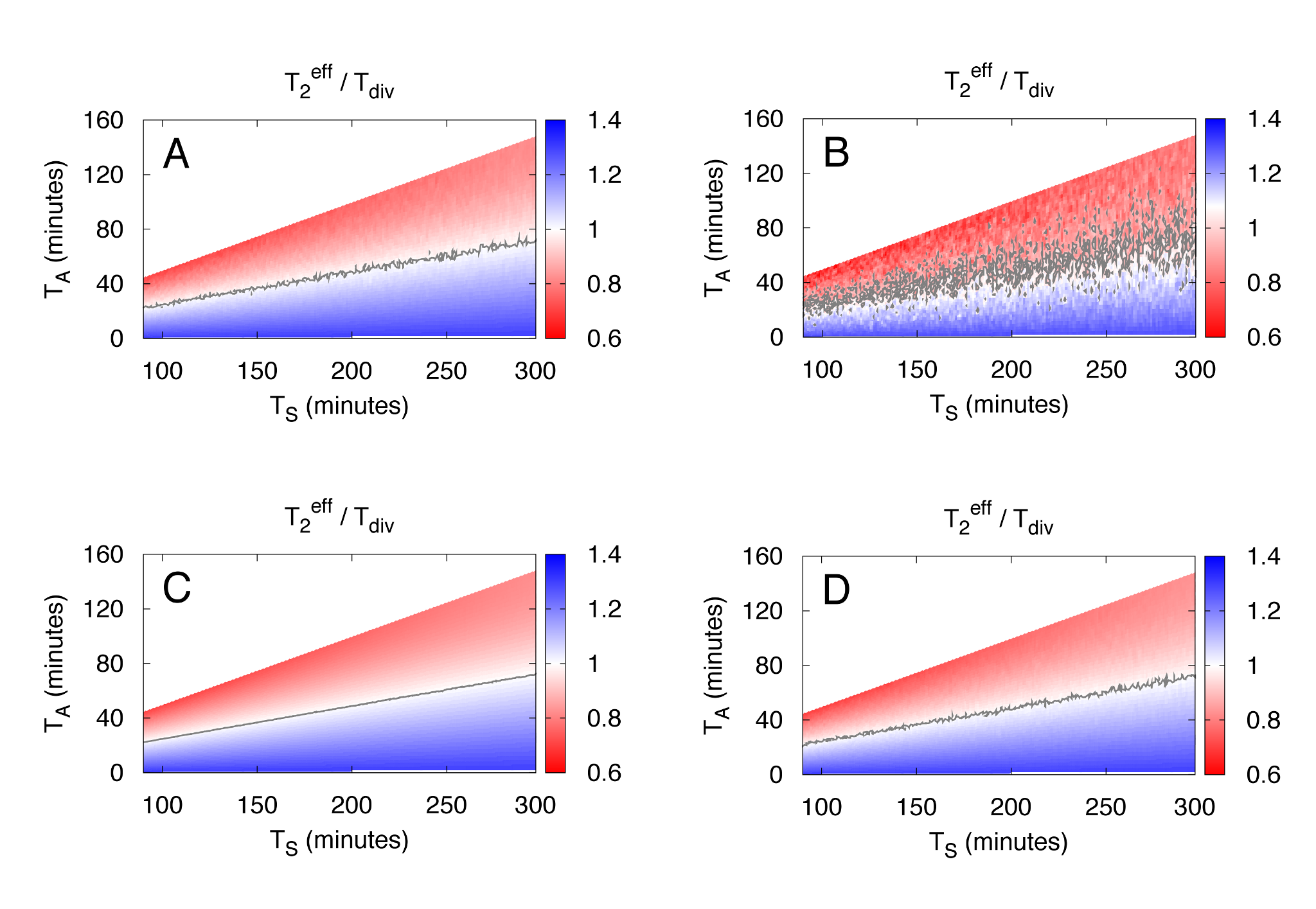

Supplement: Figure S6 — Effect of noisy switching on phenotypic diversification described by model 2. is shown as function of average switching time and adaptation time . The diversification probability is . In figures A and B, the population growth was followed for 8 switching periods. In A, switching times are drawn from a normal distribution, in B from an exponential distribution. Figures C and D show averages over 100 runs shown in A and B, respectively. Birth mass noise is 8%. We only consider environmental switches with . (TIF) [file pone.0029932.s007.tif]
